# Clinical characteristics in patients with ossification of the posterior longitudinal ligament: A prospective multi-institutional cross-sectional study

**DOI:** 10.1038/s41598-020-62278-3

**Published:** 2020-03-26

**Authors:** Takashi Hirai, Toshitaka Yoshii, Shuta Ushio, Kanji Mori, Satoshi Maki, Keiichi Katsumi, Narihito Nagoshi, Kazuhiro Takeuchi, Takeo Furuya, Kei Watanabe, Norihiro Nishida, Kota Watanabe, Takashi Kaito, Satoshi Kato, Katsuya Nagashima, Masao Koda, Kenyu Ito, Shiro Imagama, Yuji Matsuoka, Kanichiro Wada, Atsushi Kimura, Tetsuro Ohba, Hiroyuki Katoh, Yukihiro Matsuyama, Hiroshi Ozawa, Hirotaka Haro, Katsushi Takeshita, Masahiko Watanabe, Morio Matsumoto, Masaya Nakamura, Masashi Yamazaki, Atsushi Okawa, Yoshiharu Kawaguchi

**Affiliations:** 1 Department of Orthopedic Surgery, Tokyo Medical and Dental University, Tokyo, Japan; 2 Department of Orthopaedic Surgery, Shiga University of Medical Science, Tsukinowa-cho, Seta, Otsu, Shiga, 520-2192 Japan; 3 Department of Orthopedic Surgery, Chiba University Graduate School of Medicine, Chiba, Japan; 4 Department of Orthopedic Surgery, Niigata University Medical and Dental General Hospital, Niigata, Japan; 5 Department of Orthopedic Surgery, Keio University, School of Medicine, Tokyo, Japan; 6 Department of Orthopedic Surgery, National Hospital Organization Okayama Medical Center, Okayama, Japan; 7 Department of Orthopedic Surgery, Yamaguchi University Graduate School of Medicine, Yamaguchi, Japan; 8 Department of Orthopaedic Surgery, Osaka University Graduate School of Medicine, Osaka, Japan; 9 Department of Orthopedic Surgery, Graduate School of Medical Sciences, Kanazawa University, Kanazawa, Japan; 10 Department of Orthopedic Surgery, Faculty of Medicine, University of Tsukuba, Ibaraki, Japan; 11 Department of Orthopedic Surgery, Nagoya University Graduate School of Medicine, Nagoya, Japan; 12 Department of Orthopedic Surgery, Tokyo Medical University, Tokyo, Japan; 13 Department of Orthopedic Surgery, Hirosaki University Graduate School of Medicine, Hirosaki, Japan; 14 Department of Orthopedics, Jichi Medical University, Jichi, Japan; 15 Department of Orthopedic Surgery, University of Yamanashi, Yamanashi, Japan; 16 Department of Orthopedic Surgery, Surgical Science, Tokai University School of Medicine, Tokai, Japan; 17 Department of Orthopedic Surgery, Hamamatsu University School of Medicine, Hamamatsu, Japan; 18 Department of Orthopaedic Surgery, Tohoku Medical and Pharmaceutical University, Tohoku, Japan; 19 Department of Orthopedic Surgery, Faculty of Medicine, University of Toyama, Toyama, Japan; 20 Japanese Organization of the Study for Ossification of Spinal Ligament (JOSL), Toyama, Japan

**Keywords:** Spinal cord diseases, Chronic pain

## Abstract

Ossification of the posterior longitudinal ligament (OPLL) can occur throughout the entire spine and can sometimes lead to spinal disorder. Although patients with OPLL sometimes develop physical limitations because of pain, the characteristics of pain and effects on activities of daily living (ADL) have not been precisely evaluated in OPLL patients. Therefore, we conducted a multi-center prospective study to assess whether the symptoms of cervical OPLL are different from those of cervical spondylosis (CS). A total of 263 patients with a diagnosis of cervical OPLL and 50 patients with a diagnosis of CS were enrolled and provided self-reported outcomes, including responses to the Japanese Orthopaedic Association (JOA) Cervical Myelopathy Evaluation Questionnaire (JOACMEQ), JOA Back Pain Evaluation Questionnaire (JOABPEQ), visual analog scale (VAS), and SF-36 scores. The severity of myelopathy was significantly correlated with each domain of the JOACMEQ and JOABPEQ. There was a negative correlation between the VAS score for each domain and the JOA score. There were significantly positive correlations between the JOA score and the Mental Health, Bodily Pain, Physical Functioning, Role Emotional, and Role Physical domains of the SF-36. One-to-one matching resulted in 50 pairs of patients with OPLL and CS. Although there was no significant between-group difference in scores in any of the domains of the JOACMEQ or JOABPEQ, the VAS scores for pain or numbness in the buttocks or limbs were significantly higher in the CS group; however, there was no marked difference in low back pain, chest tightness, or numbness below the chest between the two study groups. The scores for the Role Physical and Body Pain domains of the SF-36 were significantly higher in the OPLL group than in the CS group, and the mean scores for the other domains was similar between the two groups. The results of this study revealed that patients with OPLL were likely to have neck and low back pain and restriction in ADL. No specific type of pain was found in patients with OPLL when compared with those who had CS.

## Introduction

Ossification of the spinal ligaments can occur throughout the entire spine and can sometimes lead to a spinal disorder. The posterior longitudinal ligament is the most common site of spinal ligament ossification and has been widely investigated worldwide. Ossification of the posterior longitudinal ligament (OPLL) can result in not only myelopathy but also spinal cord injury in asymptomatic cases following minor head trauma. Clinically, patients with OPLL sometimes develop physical limitations because of pain rather than because of motor dysfunction. Of note, compression of the spinal cord by an ossified lesion appears to be more severe than that caused by spondylosis and might be more painful. Although researchers have investigated several issues related to ossification, including prevalence^1–3^, distribution of the ossified lesions^1–6^, and neurologic outcomes after surgical treatment^7–10^, there has been no study documenting in detail the subjective symptoms and self-reported ability to perform activities of daily living (ADL) in patients with OPLL.

The aims of this study were to investigate the relationship between severity of pain and neurologic status in patients with OPLL and to identify whether there are any specific clinical symptoms in patients with cervical OPLL that are not present in those with cervical spondylosis (CS), which often leads to myelopathy similar to that occurring in cervical OPLL.

## Patients and methods

This multicenter, prospective cross-sectional study included 16 member institutions of the Japanese Multicenter Research Organization for Ossification of the Spinal Ligament formed by the Japanese Ministry of Health, Labour and Welfare. Two hundred and sixty-three Japanese patients with cervical OPLL were enrolled from September 2015 to December 2017. The inclusion criteria were presentation with symptoms including neck pain, numbness in the upper and/or lower extremities, clumsiness, and gait disturbance as well as radiographic confirmation of having cervical OPLL based on X-ray. We excluded patients who had undergone anterior decompression surgery or posterior OPLL surgery and those aged younger than 20 years.

To determine if there was any type of pain or ADL impairment specific to patients with OPLL, we compared the data for these patients with data collected for 50 patients with CS who had symptoms of neck pain, numbness in the upper and/or lower extremities, clumsiness, and gait disturbance as well as radiographic confirmation of degenerative CS.

The study protocol was approved by the institutional review board of each participating institution and performed in accordance with the relevant guidelines and regulations. Informed consent was obtained from all patients before enrollment in the study.

### Evaluations

Basic demographic and clinical data, including patient age and sex, presence of diabetes, body mass index (BMI), and presence of neck pain, back pain, and low back pain were collected for each patient. Clinical status was evaluated using the cervical Japanese Orthopedic Association (JOA) score (Supplemental Table 1)^11^, JOA Cervical Myelopathy Evaluation Questionnaire (JOACMEQ, Supplemental Table 2)^12^, JOA Back Pain Evaluation Questionnaire (JOABPEQ, Supplemental Table 3)^12^, and the 36-item Short Form Health Survey (SF-36)^13^. A visual analog scale (VAS) was used to evaluate the degree of pain or stiffness in the neck or shoulders, tightness in the chest, pain or numbness in the arms or hands, pain or numbness from the chest to the toes, low back pain, and pain or numbness in the buttocks and lower limbs (Supplemental Table 4). The patients were informed that numbness was defined as subjective paresthesia, hypersensitivity, and hyperesthesia. In addition, types of OPLL patients were classified into ‘continuous’ type and ‘other’ types (mixed, segmental, and other) based on plain lateral X-ray in order to compare rigid and motion-preserved cervical OPLL.

### Statistical analysis

The two groups of patients were compared using the Student’s unpaired *t*-test, Mann Whitney *U* test, and the chi-squared test. Propensity score matching was performed for age, sex, BMI, JOA score, and whether or not diabetes mellitus was present. Propensity scores were generated by a multivariable logistic regression model using SPSS for Windows version 22.0 (IBM Corp., Armonk, NY) to compare the physical status of the patients with OPLL with that of those with CS. All the study data are shown as the mean ± standard deviation. A *p*-value <0.05 was considered statistically significant.

### Ethics approval and consent to participate

Written informed consent was obtained from each study participant before enrollment at each institution. The study protocol was approved by the ethics committee at each participating institution i.e., Shiga University of Medical Science, Tokyo Medical and Dental University, Keio University, National Hospital Organization Okayama Medical Center, University of Toyama, Hirosaki University Graduate School of Medicine, Chiba University Graduate School of Medicine, Jichi Medical University, University of Yamanashi, Tokai University School of Medicine, Niigata University Medicine and Dental General Hospital, Tokyo Medical University, Tohoku Medical and Pharmaceutical University Tohoku University School of Medicine, Nagoya University Graduate School of Medicine, Kyoto University, and University of Tsukuba.

## Results

### Demographics in the study groups

In total, 263 patients with OPLL (177 male, 86 female; mean age 63.6 years) and 50 with CS (33 male, 17 female; mean age 67.9 years) were enrolled. The patient demographics are shown in Table 1. The mean BMI was 27.1 in the OPLL group and 23.7 in the CS group. Comorbid diabetes mellitus was present in 24.7% of patients with OPLL and in 30% of those with CS. The patients with CS were significantly older than those with OPLL.

**Table 1:** Comparison of demographic data at baseline.

	OPLL group n = 263	CS group n = 50	*p*-value
Age, years	63.6 ± 12.3	67.9** ± 10.6	0.01
Sex (Male: Female)	177: 86	33: 17	0.87
Height, cm	162.9 ± 10.1	159.1** ± 9.6	0.01
Body weight, kg	72.0 ± 47.2	60.9 ± 11.0	0.08
Comorbid diabetes mellitus	65 (24.7%)	15 (30%)	0.46
**Cervical myelopathic JOA score**			
Upper extremity motor function	2.9 ± 1.0	2.3*** ± 1.1	<0.001
Lower extremity motor function	2.7 ± 1.2	2.2*** ± 1.2	<0.001
Sensory function in the upper extremities	1.2 ± 0.6	1.1 ± 0.5	0.17
Sensory function in the trunk	1.7 ± 0.5	1.9 ± 0.4	0.06
Sensory function in the lower extremities	1.4 ± 0.6	1.5 ± 0.5	0.40
Bladder function	2.5 ± 0.7	2.6 ± 0.7	0.33
Total	12.4 ± 3.4	11.4* ± 3.4	0.04
**Complaint**			
Neck pain	161 (61.2%)	47*** (94%)	<0.001
Back pain	75 (28.5%)	15 (30%)	0.87
Low back pain	138 (52.4%)	39*** (78%)	<0.001
**JOA-CMEQ**			
Cervical spine function	65.6 ± 29.2	69.7 ± 26.6	0.35
Upper extremity function	80.4 ± 21.1	73.9* ± 22.9	0.04
Lower extremity function	66.6 ± 30.2	57.1* ± 28.2	0.04
Bladder function	75.5 ± 21.6	74.0 ± 20.1	0.66
Quality of life	50.2 ± 19.7	46.1 ± 19.8	0.15
**JOA-BPEQ**			
Lumbar spine function	68.7 ± 31.4	64.7 ± 31.4	0.38
Social life function	56.9 ± 29.2	53.3 ± 28.1	0.32
Walking ability	64.5 ± 35.1	53.3* ± 36.4	0.03
Body pain	70.8 ± 34.2	70.8 ± 37.4	0.90
Mental health	49.2 ± 19.8	48.4 ± 19.7	0.73
**VAS scores**			
Neck or shoulder pain or numbness	39.1 ± 30.8	49.6* ± 30.8	0.02
Chest tightness	10.2 ± 21.4	7.9 ± 18.6	0.45
Upper extremity pain or numbness	44.2 ± 32.9	62.6*** ± 32.5	<0.001
Pain or numbness below the chest	37.3 ± 33. 7	41.6 ± 34.8	0.37
Low back pain	27.3 ± 28.7	33.1 ± 25.8	0.16
Pain in lower extremities	23.1 ± 29.9	35.7* ± 33.8	0.02
Numbness in lower extremities	30.6 ± 33.4	33.0 ± 31.8	0.35
**SF36 scores**			
Physical Functioning	60.8 ± 30.5	54.5 ± 28.9	0.16
Role Physical	58.0 ± 32.3	46.5** ± 28.6	0.01
Bodily Pain	51.6 ± 25.8	40.3*** ± 23.4	<0.001
General Health	49.3 ± 8.9	47.7 ± 7.8	0.27
Vitality	56.5 ± 12.9	58.9 ± 14.0	0.27
Social Functioning	51.5 ± 12.5	53.0 ± 14.2	0.39
Role Emotional	64.6 ± 32.8	51.0*** ± 30.2	<0.001
Mental Health	60.2 ± 11.9	61.5 ± 11.4	0.61

Data are shown as the mean and standard deviation or as the number (percentage) as appropriate. BPEQ, Back Pain Evaluation Questionnaire; CMEQ, Cervical Myelopathy Evaluation Questionnaire; CS, cervical spondylosis; JOA, Japanese Orthopaedic Association; OPLL, ossification of the posterior longitudinal ligament; SF-36, Short Form-36; VAS, visual analog scale. *Weakly significant difference (0.01 < p < 0.05); **moderately significant difference (0.001 < p < 0.01); ***highly significant difference (p < 0.001).

The mean JOA cervical myelopathy score was 12.4 in the patients with OPLL and 11.4 in those with CS, indicating more severe myelopathy in the CS group. Neck pain was reported by 61.2% of the patients with OPLL and 94% of those with CS, back pain by 28.5% and 30%, respectively, and low back pain by 52.4% and 78%. The prevalence rates of neck pain and low back pain were higher in the patients with CS.

The mean scores for the various JOACMEQ domains were as follows: 65.6 in patients with OPLL and 69.7 in those with CS for cervical function, 80.4 and 73.9, respectively, for upper extremity function, 66.6 and 57.1 for lower extremity function, 75.5 and 74.0 for bladder function, and 50.2 and 46.1 for quality of life. The mean JOABPEQ score was 68.7 in patients with OPLL and 64.7 in those with CS for lumbar function, 56.9 and 53.3 for social participation, 64.5 and 53.3 for walking ability, 70.8 and 70.8 for pain, and 49.2 and 48.4 for mental health. Therefore, the scores were significantly lower for functioning of the upper and lower extremities and walking ability in patients with CS.

The mean VAS score was 39.1 in the OPLL group and 49.6 in the CS group for pain or stiffness in the neck or shoulder, 10.2 and 7.9, respectively, for tightness in the chest, 44.2 and 62.6 for pain or numbness in the arms or hands, 37.3 and 41.6 for pain or numbness from the chest to the toes, 27.3 and 33.1 for low back pain, 23.1 and 35.7 for pain in the buttocks and lower limbs, and 30.6 and 33.0 for numbness in the buttocks and lower limbs. The between-group differences in the VAS scores for neck and shoulder pain or numbness, pain or numbness in the upper extremities, and numbness in the lower extremities were statistically significant.

The mean scores for each domain of the SF-36 were as follows: 60.8 in patients with OPLL and 54.5 in those with CS for Physical Functioning, 58.0 and 46.5, respectively, for Role Physical, 51.6 and 40.3 for Bodily Pain, 49.3 and 47.7 for General Health, 56.5 and 58.9 for Vitality, 51.5 and 53.0 for Social Functioning, 64.6 and 51.0 for Role Emotional, and 60.2 and 61.5 for Mental Health. These results indicate that patients with CS had significant poorer scores in the Role Physical, Bodily Pain, and Role Emotional domains than those with OPLL.

### Significant associations between the neurologic status of patients with OPLL and body function, ADL, and pain

**Figure 1:**
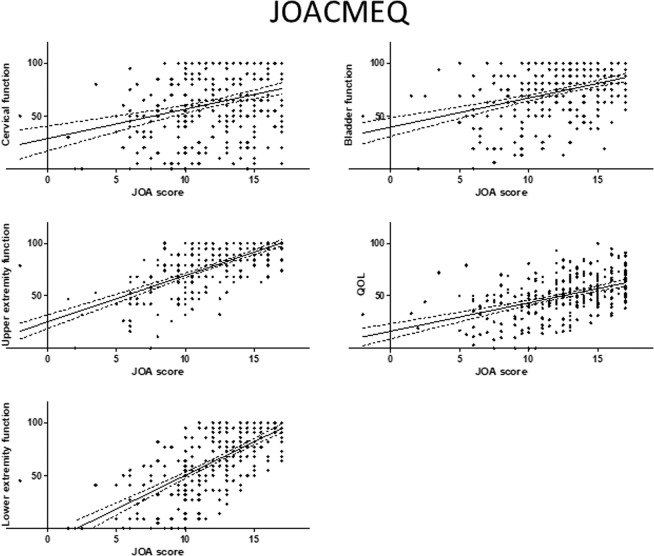
Relationship between severity of myelopathy and each domain of the JOACMEQ. The JOA score for myelopathy was significantly correlated with the score on each domain of the JOACMEQ. JOA, Japanese Orthopedic Association; JOACMEQ, Japanese Orthopedic Association Cervical Myelopathy Evaluation Questionnaire.

**Figure 2:**
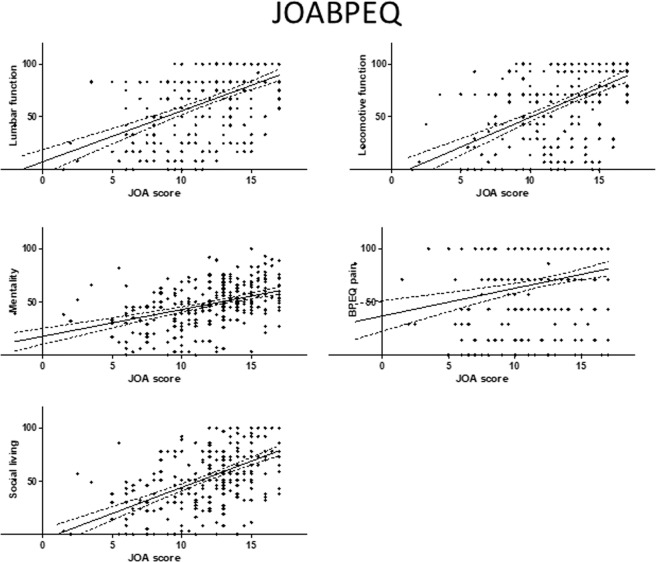
Relationship between severity of myelopathy and each domain of the JOABPEQ. The JOA score for myelopathy was significantly correlated with the score on each domain of the JOABPEQ. JOA, Japanese Orthopedic Association; JOABPEQ, Japanese Orthopedic Association Back Pain Evaluation Questionnaire.

**Figure 3:**
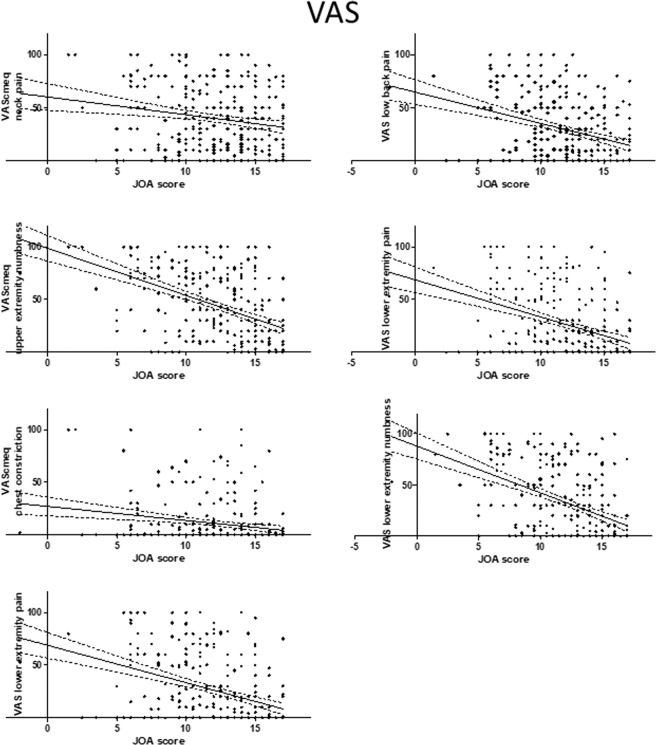
Relationship between severity of myelopathy and each domain of the VAS. The VAS score for each domain of the VAS was negatively correlated with the JOA score for myelopathy. JOA, Japanese Orthopedic Association; VAS, visual analog scale.

**Figure 4:**
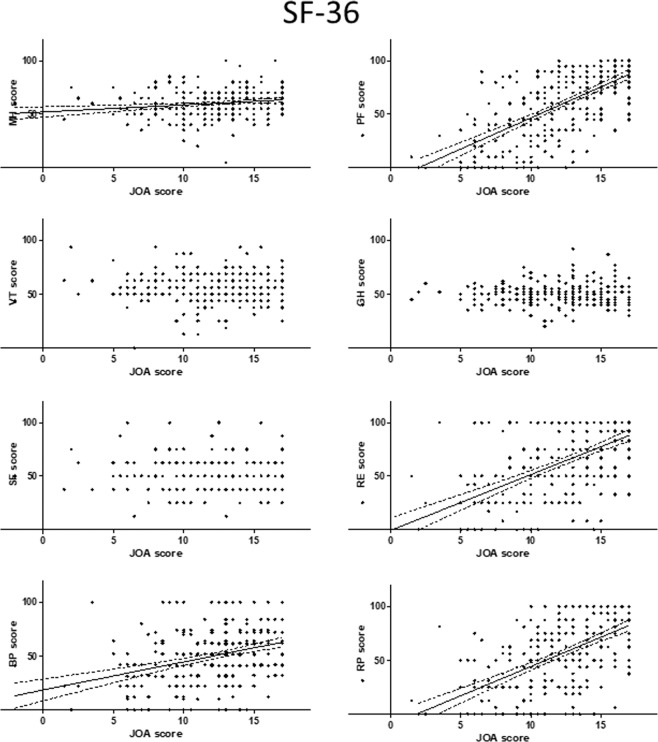
Relationship between severity of myelopathy and each domain of the SF-36. There were significant positive correlations between the JOA score for myelopathy and MH, BP, PF, RE, and RP, respectively. BP, Bodily Pain; MH, Mental Health; PF, Physical Functioning; RE, Role Emotional; RP, Role Physical; SF-36, 36-item Short Form Health Survey.

The relationships between the JOA score and patient-reported evaluations were investigated to confirm whether or not neurologic symptoms were associated with body functioning in patients with OPLL. Overall, the JOA score was significantly correlated with the score on each domain of the JOACMEQ (Fig. 1) and JOABPEQ (Fig. 2). The VAS score for each domain was negatively correlated with the JOA score (Fig. 3). There were significant positive correlations of the JOA score with the Mental Health, Bodily Pain, Physical Functioning, Role Emotional, and Role Physical domains of the SF-36 (Fig. 4).

### Less body pain in patients with continuous OPLL than in patients with other types of OPLL

Out of 263 cases, radiologic data could be obtained from 239 patients. To investigate whether different types of OPLL have different types of pain or restriction of ADL in OPLL patients, these 239 patients were divided into continuous OPLL (n = 17) and other types of OPLL, namely segmental, mixed, and other (n = 222). Although there were no significant differences in terms of basic data, the prevalence of neck pain and low back pain were significantly lower in patients with continuous OPLL compared with patients with other types (Table 2). With regards to the JOACMEQ and JOABPEQ scores, no significant difference was observed between the two arms. However, patients with continuous OPLL reported less pain or numbness in the lower extremities based on VAS scores compared with cases with other types of OPLL. In addition, there was no significant difference in each score of the SF36, excerpt for the Bodily pain domain.

**Table 2:** Comparison between patients with continuous OPLL and patients with other types of OPLL.

	Continuous OPLL n = 17	Other types of OPLL n = 222	p-value
Age, years	69.4 ± 12.1	63.5 ± 12.2	0.27
Sex (Male: Female)	12: 5	151: 71	0.83
Height, cm	163.3 ± 9.5	162.8 ± 9.8	0.71
Body weight, kg	63.3 ± 13.2	69.6 ± 15.5	0.71
Comorbid diabetes mellitus (n, rate)	4 (23.5%)	55 (24.8%)	0.91
Cervical myelopathic JOA score	11.8 ± 3.8	12.3 ± 3.4	0.17
**Complains**			
Neck pain	6 (35.3%)	135 (60.8%)*	0.04
Back pain	4 (23.5%)	63 (28.4%)	0.67
Low back pain	5 (29.4%)	123 (55.4%) *	0.03
**JOA-CMEQ**			
Cervical spine function	54.1 ± 25.2	66.8 ± 28.6	0.06
Upper extremity function	71.8 ± 22.0	80.7 ± 21.4	0.12
Lower extremity function	62.5 ± 30.7	66.3 ± 30.8	0.64
Bladder function	76.7 ± 22.7	74.3 ± 22.0	0.67
Quality of life	50.2 ± 19.6	49.9 ± 20.0	0.94
**JOA-BPEQ**			
Lumbar spine function	74.9 ± 28.2	67.5 ± 31.9	0.31
Social life function	61.6 ± 28.7	55.9 ± 29.5	0.44
Walking ability	72.6 ± 30.0	63.5 ± 35.6	0.24
Body pain	84.8 ± 27.0	69.7 ± 33.9*	0.04
Mental health	42.9 ± 24.1	49.6 ± 19.6	0.27
**VAS scores**			
Neck or shoulder pain or numbness	32.6 ± 36.3	39.3 ± 30.8	0.47
Chest tightness	7.9 ± 17.0	10.4 ± 22.1	0.57
Upper extremity pain or numbness	45.0 ± 38.2	45.1 ± 33.0	0.97
Pain or numbness below the chest	23.6 ± 35.8	38.0 ± 33.8	0.13
Low back pain	15.9 ± 25.3	28.6 ± 29.1	0.06
Pain in lower extremities	7.2 ± 12.5	24.4 ± 30.8***	<0.001
Numbness in lower extremities	14.9 ± 24.9	32.2 ± 34.1*	0.01
**SF36 scores**			
Physical Functioning	60.0 ± 32.9	60.7 ± 30.8	0.94
Role Physical	59.0 ± 31.3	57.1 ± 33.0	0.80
Bodily Pain	65.3 ± 23.3	50.6 ± 26.1*	0.02
General Health	48.6 ± 8.7	49.2 ± 8.7	0.79
Vitality	60.3 ± 14.3	56.3 ± 12.9	0.27
Social Functioning	47.8 ± 11.9	51.9 ± 13.0	0.20
Role Emotional	65.1 ± 33.1	64.1 ± 33.5	0.90
Mental Health	60.3 ± 10.7	60.4 ± 11.6	0.97

Data are shown as the mean and standard deviation or as the number (percentage) as appropriate. CS, cervical spondylosis; JOA, Japanese Orthopaedic Association; OPLL, ossification of posterior longitudinal ligament.

### Between-group comparisons after propensity score matching

**Figure 5:**
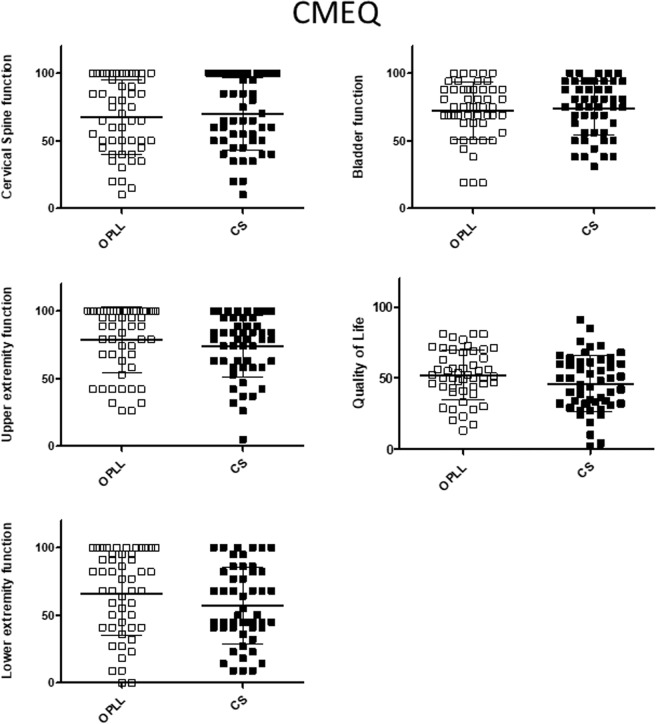
Comparison of each domain in the JOACMEQ after adjustment by propensity score matching between the study groups. There was no statistically significant difference in the JOACMEQ score between the OPLL and CS groups. CS, cervical spondylosis; JOACMEQ, Japanese Orthopedic Association Cervical Myelopathy Evaluation Questionnaire: OPLL, ossification of the posterior longitudinal ligament.

**Figure 6:**
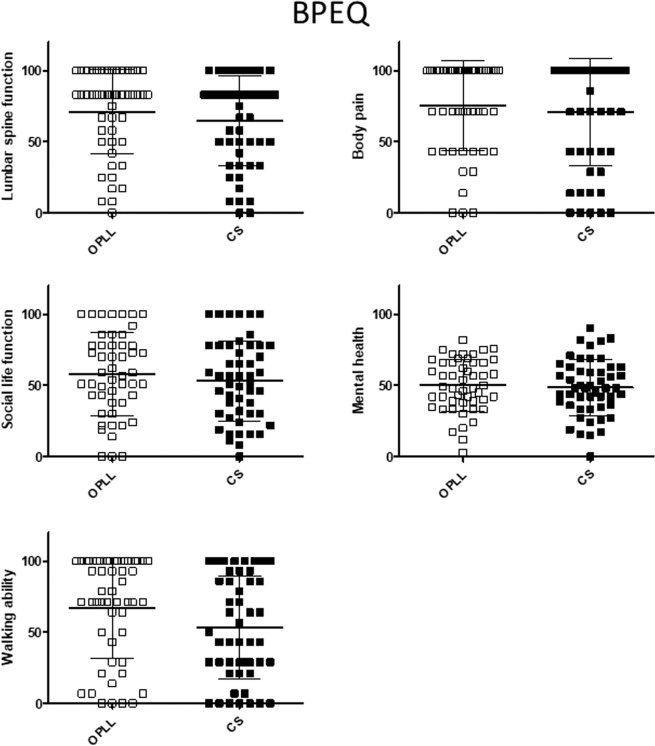
Comparison of each domain in the JOABPEQ after adjustment by propensity score matching between the study groups. There were no statistically significant between-group differences in any of the domains of the JOABPEQ. Japanese Orthopedic Association Back Pain Evaluation Questionnaire.

**Figure 7:**
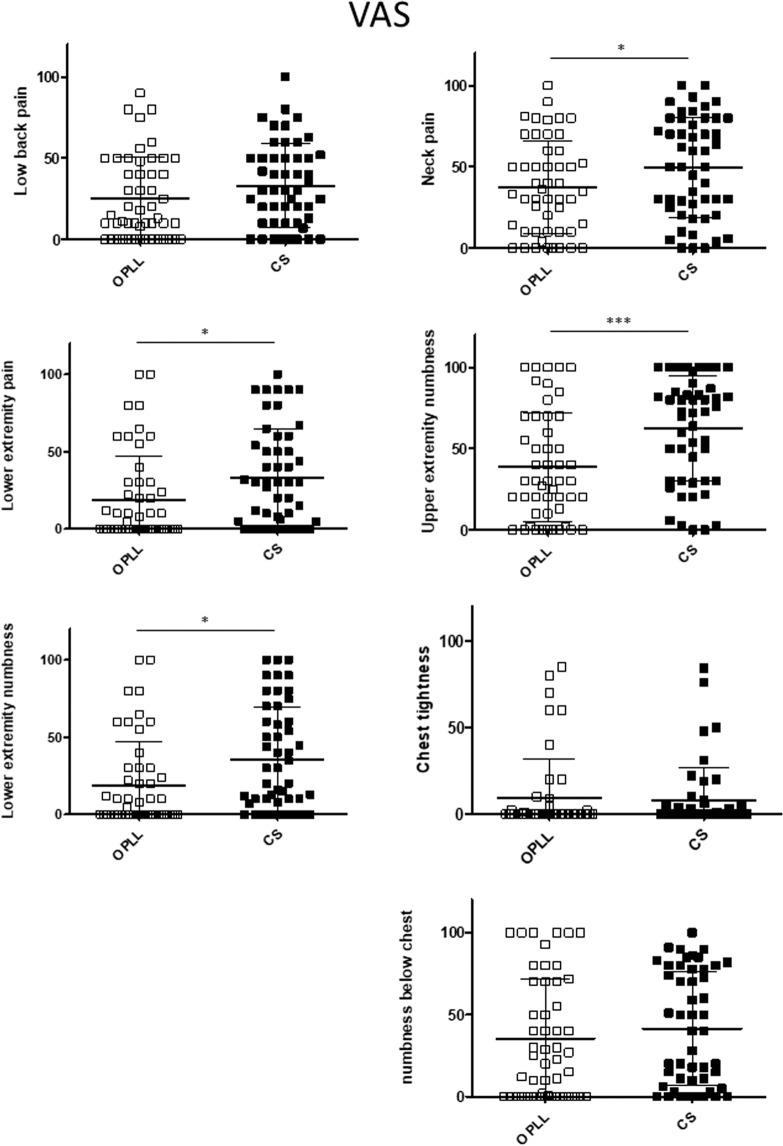
Comparison of each item on the VAS between the study groups after adjustment by propensity score matching. The VAS scores for pain or numbness in the buttocks and limbs were significantly higher in the CS group than in the OPLL group but there was no marked between-group difference in low back pain, chest tightness, or numbness below the chest. CS, cervical spondylosis; OPLL, ossification of the posterior longitudinal ligament; VAS, visual analog scale.

**Figure 8:**
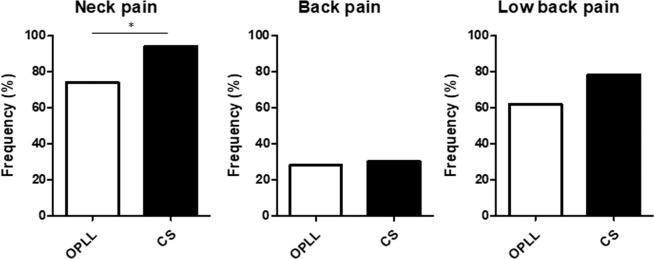
Proportion of patients with each symptom. Patients with CS were more likely to have neck pain than those with OPLL but not back pain or low back pain. CS, cervical spondylosis; OPLL, ossification of the posterior longitudinal ligament.

**Figure 9:**
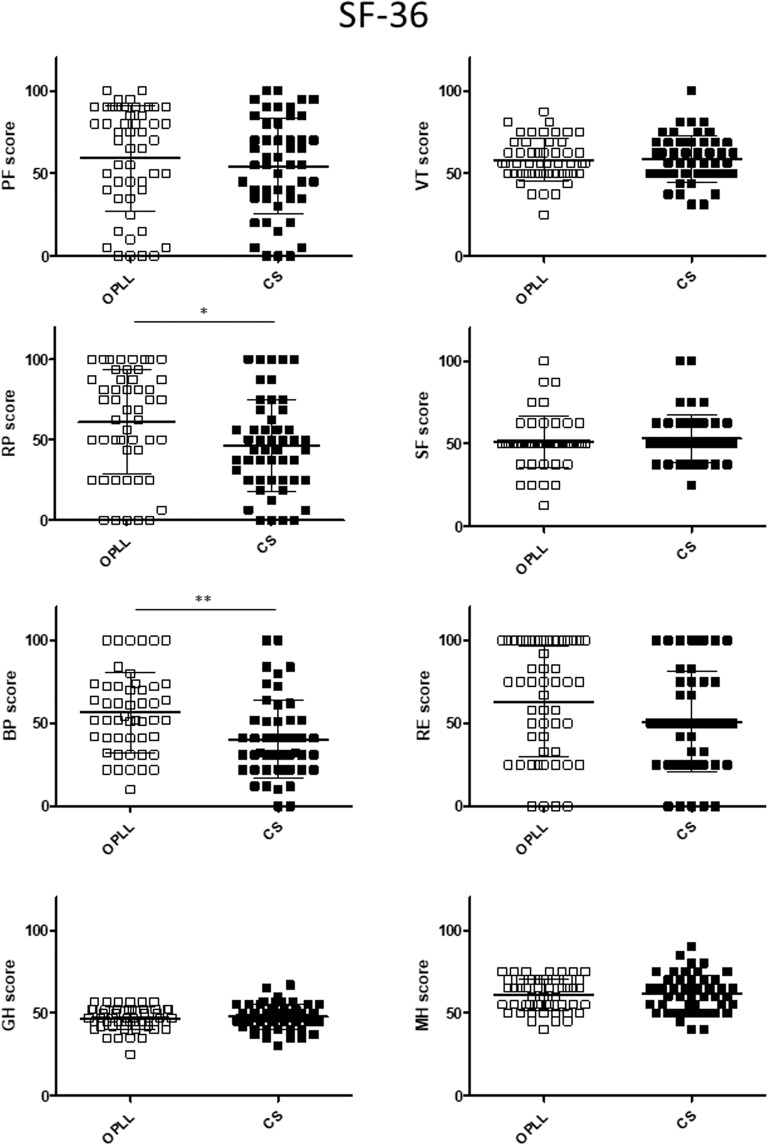
Comparison of each item in the SF-36 between the study groups after adjustment by propensity score matching. RP and BP scores were significantly higher in the OPLL group than in the CS group. CS, cervical spondylosis; BP, Bodily Pain; OPLL, ossification of the posterior longitudinal ligament; RP, Role Physical.

All patients were included in the propensity score calculation because of differences in the baseline demographic data between the study groups. The c-statistic was 0.75 (95% confidence interval 0.687–0.812). One-to-one matching resulted in 50 pairs of patients with OPLL and CSM (Table 3). The distribution of propensity scores is demonstrated in histograms before and after matching in Supplemental Figs. 1 and 2. There was no significant between-group difference in any domain of the JOACMEQ or JOABPEQ (Figs. 5 and 6). The VAS scores for pain or numbness in the buttocks or limbs were significantly higher in the CS group than in the OPLL group; however, there were no marked between-group differences in reports of low back pain, chest tightness, or numbness below the chest (Fig. 7). The proportion of patients who reported neck pain (but not back pain or low back pain) was significantly higher in the CS group than in the OPLL group (Fig. 8). The scores for the Role Physical and Bodily Pain domains of the SF-36 were significantly higher in the OPLL group than in the CS group; however, the average scores in the other domains was similar between the two groups (Fig. 9).

**Table 3:** Comparison of propensity score-matched data between the study groups at baseline.

	OPLL group n = 50	CS group n = 50	*p*-value
Age, years	69.3 ± 9.9	67.9 ± 10.6	0.53
Sex (Male: Female)	33: 17	33: 17	1
Height, cm	158.4 ± 7.8	159.1 ± 9.6	0.71
Body weight, kg	61.0 ± 9.1	60.9 ± 11.0	0.97
Comorbid diabetes mellitus	15 (30%)	15 (30%)	1
Cervical myelopathic JOA score	12.1 ± 3.8	11.4 ± 3.4	0.35

Data are shown as the mean and standard deviation or as the number (percentage) as appropriate. CS, cervical spondylosis; JOA, Japanese Orthopaedic Association; OPLL, ossification of the posterior longitudinal ligament.

## Discussion

OPLL was first described in the 1960s^14^ and is now understood to result from heterotopic bone formation in the posterior longitudinal ligament of the spine and to be a common cause of myelopathy, especially in Asia. Although several researchers have investigated the symptoms of OPLL and the efficacy of surgery, there are few reports on pain and impairment of ADL in patients with this disorder^15,16^. Sasaki *et al*. reviewed a sample (n = 1291) of the general population in Japan and divided it into subjects with and without OPLL^15^. They found that 86.7% of patients in the female subjects with OPLL group had symptoms and that female subjects in the OPLL group had more severe neck pain than their counterparts without OPLL. Given that the subjects in that study were recruited from the general population, the symptoms in subjects with OPLL might have been milder than those in patients with OPLL who presented to hospital, as in this study, which specifically recruited symptomatic patients with OPLL.

Although the JOA cervical score has been validated in patients with myelopathy, the main focus when it has been used in OPLL studies has been on motor dysfunction rather than sensory deficit or pain. However, patients with OPLL often complain of body pain and receive medication for their subjective symptoms. Therefore, it is important to understand the extent of pain and recognize deterioration of ADL in patients with OPLL. We believe that our study is the first multi-institutional investigation of self-reported ADL, quality of life, and pain in this patient population.

Our study shows that patients with cervical OPLL and poor neurologic status are more likely to have whole body pain and progression of neuropathic pain as a result of spinal disorder with compression of ossified lesions. Our findings suggest that neurologic dysfunction could worsen in patients with relatively severe body pain, even in the early phase of myelopathy. Therefore, the extent of body pain may be a predictor of deterioration of myelopathy in patients with OPLL.

A nationwide survey of the general population reported that the average score for each domain of the JOACMEQ, except for quality of life, was almost 100 points^17^. However, in this study, we found that patients with cervical OPLL had unsatisfactory scores in all domains of the JOACMEQ. We also found a similar trend in the JOABPEQ. Despite the higher likelihood of those in their 80 s having a worse score, the mean score for each domain, except for mental health, was over 80 in the general population^17^. In our study, patients with cervical OPLL had poor scores, even for functioning of the lumbar spine. Furthermore, the average score in each section of the SF-36 was 50–65 points.

The severity of myelopathy in patients with OPLL does not always correlate with scores in the Vitality, Social Functioning, and General Health domains of the SF-36. However, the mean scores for these items were significantly lower in patients with OPLL than in healthy volunteers. Nakajima *et al*. reviewed 39 patients with OPLL and spinal cord-induced chronic pain and also found that their scores for all items on the SF-36 were significantly lower than the national average^18^. Therefore, care is needed in patients with OPLL, who have impaired ability to perform ADL because of motor dysfunction and/or pain.

A cross-sectional observational cohort study of healthy volunteers demonstrated that the prevalence rates of both low back pain and neck pain were approximately 10% in the Japanese population^17^. Our findings indicate that among patients with OPLL, those with severe symptoms likely have more pain. Interestingly, we found a strong association between the severity of myelopathy and low back pain, which was not directly related to the pathology of cervical OPLL and has not been reported before. Takenaka *et al*. reviewed 205 patients with thoracic myelopathy who underwent surgery and performed multivariate analyses to identify factors that were associated with pain^19^. They found that anterior compression caused by OPLL was a more significant determinant of low back pain and lower limb pain in the thoracolumbar spine than ossification of the ligament flavum or intervertebral herniation. A retrospective multicenter study that included patients with cervical OPLL reported that OPLL was also present in the thoracolumbar spine in 17.8% of cases and in the lumbar spine in 12.1%^20^. Furthermore, it has been documented that the prevalence of OPLL in the thoracolumbar spine increases with the number of cervical levels affected by OPLL^1,2^. Similarly, patients with CS often have low back pain as well. Yamada *et al*. demonstrated that cervical spondylotic stenosis in patients without OPLL often coexists with lumbar canal stenosis and sometimes requires decompressive surgery^21^. Overall, our study finding suggests that patients with and without cervical OPLL may have multiple lesions in the thoracolumbar spine and thus be more likely to develop low back pain and symptoms in the lower extremities.

In this study, we compared a group of patients with cervical OPLL with a group that had CS to determine whether or not there are specific symptoms or types of pain that occur in patients with OPLL. Propensity score matching was performed before comparing the clinical features of the patients with OPLL and those with CS because of between-group differences in demographic data. Although this analysis revealed no significant difference in neurologic function as evaluated by the JOABPEQ and JOACMEQ between the two arms, the VAS scores indicated that patients in the CS group were more likely to complain of neck pain, numbness in all four limbs, and pain in the lower extremities. Fujimori *et al*. compared a group of patients with OPLL and a group of patients with cervical spondylotic myelopathy and reported that the VAS scores for neck pain and arm symptoms were higher in the group with cervical spondylotic myelopathy^22^. Nakajima *et al*. also reported that spontaneous neuropathic pain and paresthesia scores were higher in patients with cervical spondylotic myelopathy than in those with OPLL^18^. Our results are consistent with their finding that CS is more painful than OPLL. Given that patients with CS have been found to be more likely to have neck and/or upper extremity pain as a result of cervical radiculopathy, we speculate that the mean intensity of neck and upper extremity pain would be greater in patients with CS than in those with cervical OPLL. Interestingly, we found that patients with continuous OPLL were less likely have neck pain or low back pain compared with those with other types of ossification in which the cervical spine has more mobility than in continuous OPLL. It has also been documented that unstable segmental motion in the cervical spine can impair neurologic function and cause pain^23,24^, probably because mobility of the cervical spine is likely to be more restricted in patients with OPLL than in those with spondylosis^25^. These findings and evidence could account for why patients with CS or motion preserved-OPLL have higher pain scores for the neck and all four limbs.

Whether or not there is an association between the radiologic severity of the ossified lesion and pain remains unclear. Some studies have reported no significant difference in neck pain and arm pain between cervical OPLL patients with and without intramedullary signal intensity changes on T2-weghted magnetic resonance imaging. However, radiologic findings have been considered to be a significant factor in severe symptoms of myelopathy such as motor and posterior column dysfunction^18,26–28^. Further evaluation of imaging features, including the degree of spinal cord compression, mobility of the spinal segment affected, and changes in signal intensity of the cord, will be required to clarify the relationship between pain in patients with cervical OPLL and radiologic findings.

This study has several limitations. First, although the study was it was conducted prospectively, it was not population-based. Second, only one-sixth of the patients with OPLL were included in the final analysis after propensity score matching. Third, we did not investigate the relationship between severity of symptoms and detailed radiologic findings, such as occupancy rate or degree of ankylosis, in patients with OPLL or the compressive lesions in those with CS based on computed tomography in the whole spine. Finally, the study had a cross-sectional design, and therefore a longitudinal investigation will be needed to validate the association between severity of myelopathy and body pain. However, despite these limitations, we believe that our findings provide important information and insights concerning the diagnostic features of patients with OPLL and those with spondylosis.

## Conclusion

This is the first well-powered, multicenter prospective study to identify that patients with OPLL are likely to have neck and low back pain and impairment of ADL. This study also found that there is no specific type of pain in patients with OPLL that distinguishes them from those with cervical spondylosis. More studies are needed to investigate the underlying pathophysiology and reasons for these significant findings to shed further light on the neurologic status of patients with OPLL.

## Supplementary information

Supplementary Information.

## Data Availability

The study data and details of materials used may be made available upon reasonable request by sending an e-mail to the first author.
