# Supplementary material for: Clinical characteristics in patients with ossification of the posterior longitudinal ligament: A prospective multi-institutional cross-sectional study
Source: Sci Rep. 2020 Mar 26;10:5532. doi: 10.1038/s41598-020-62278-3 (PMC7099083; doi:10.1038/s41598-020-62278-3)
Supplement: Supplementary file 1 — Supplementary Information. [file 41598_2020_62278_MOESM1_ESM.pdf]

Clinical characteristics in patients with ossification of the posterior longitudinal ligament: A prospective multi-institutional cross-sectional study

Takashi Hirai, MD,[1, 20] Toshitaka Yoshii, MD,[1, 20] Shuta Ushio, MD,[1, 20] Kanji Mori, MD,[2,20] Satoshi Maki, MD,[3, 20] Keiichi Katsumi, MD,[4, 20] Narihito Nagoshi, MD,[5, 20] Kazuhiro Takeuchi, MD,[6, 20] Takeo Furuya, MD,[3, 20] Kei Watanabe, MD,[4, 20] Norihiro Nishida, MD,[7, 20] Kota Watanabe, MD,[5, 20] Takashi Kaito, MD,[8, 20] Satoshi Kato, MD,[9, 20] Katsuya Nagashima, MD,[10, 20] Masao Koda, MD,[10, 20] Kenyu Ito, MD,[11, 20] Shiro Imagama, MD,[11, 20] Yuji Matsuoka, MD,[12, 20] Kanichiro Wada, MD,[13, 20] Atsushi Kimura, MD,[14, 20] Tetsuro Ohba, MD,[15, 20] Hiroyuki Katoh, MD,[16, 20] Yukihiro Matsuyama, MD,[17, 20] Hiroshi Ozawa, MD,[18, 20] Hirotaka Haro, MD,[15, 20] Katsushi Takeshita, MD,[14, 20] Masahiko Watanabe, MD,[16, 20] Morio Matsumoto, MD,[5, 20] Masaya Nakamura, MD,[5, 20] Masashi Yamazaki, MD,[10, 20] Atsushi Okawa, MD,[1, 20] and Yoshiharu Kawaguchi, MD [19, 20]

[1] Department of Orthopedic Surgery, Tokyo Medical and Dental University
[2] Department of Orthopaedic Surgery, Shiga University of Medical Science, Tsukinowa-cho, Seta, Otsu, Shiga 520-2192 Japan.
[3] Department of Orthopedic Surgery, Chiba University Graduate School of Medicine
[4] Department of Orthopedic Surgery, Niigata University Medical and Dental General Hospital
[5] Department of Orthopedic Surgery, Keio University, School of Medicine
[6] Department of Orthopedic Surgery, National Hospital Organization Okayama Medical Center
[7] Department of Orthopedic Surgery, Yamaguchi University Graduate School of Medicine
[8] Department of Orthopaedic Surgery, Osaka University Graduate School of Medicine
[9] Department of Orthopedic Surgery, Graduate School of Medical Sciences, Kanazawa University
[10] Department of Orthopedic Surgery, Faculty of Medicine, University of Tsukuba
[11] Department of Orthopedic Surgery, Nagoya University Graduate School of Medicine
[12] Department of Orthopedic Surgery, Tokyo Medical University
[13] Department of Orthopedic Surgery, Hirosaki University Graduate School of Medicine
[14] Department of Orthopedics, Jichi Medical University
[15] Department of Orthopedic Surgery, University of Yamanashi

[16] Department of Orthopedic Surgery, Surgical Science, Tokai University School of Medicine
[17] Department of Orthopedic Surgery, Hamamatsu University School of Medicine
[18] Department of Orthopaedic Surgery, Tohoku Medical and Pharmaceutical University
[19] Department of Orthopedic Surgery, Faculty of Medicine, University of Toyama
[20] Japanese Organization of the Study for Ossification of Spinal Ligament (JOSL)

Correspondence: Takashi Hirai

Department of Orthopedic Surgery, Tokyo Medical and Dental University,

1-5-45 Yushima, Bunkyo-Ward, Tokyo 113-8519, Japan.

Tel: +81-35803-5279; Fax: +81-3580-5281; E-mail: hirai.orth@tmd.ac.jp

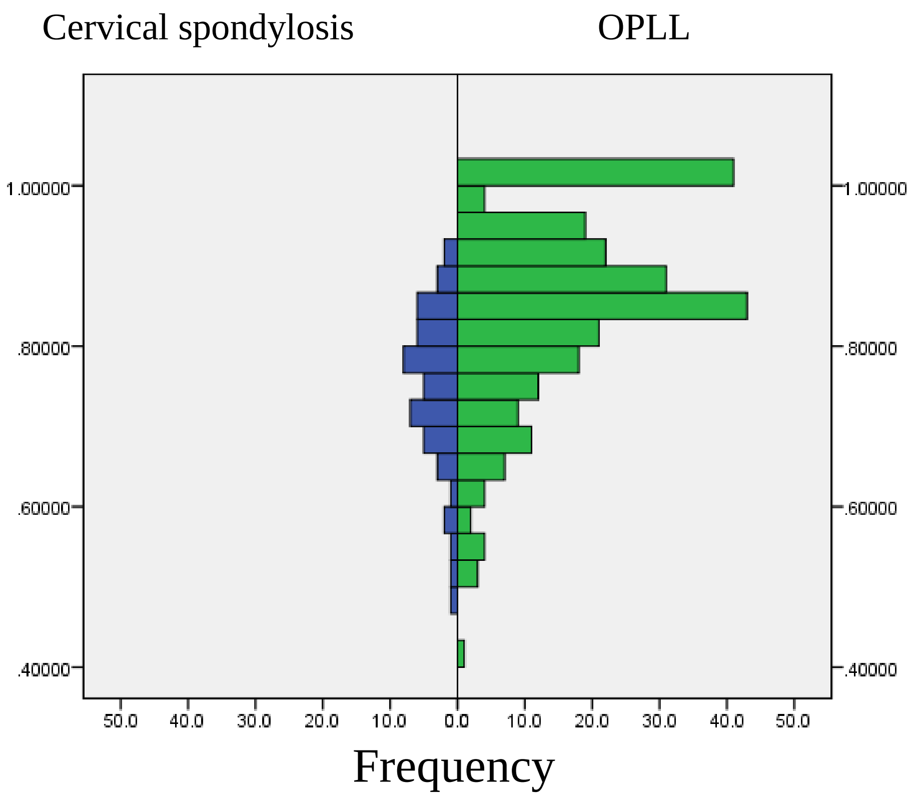

Supplementary figure 1. Histogram of propensity scores in the entire cohort.

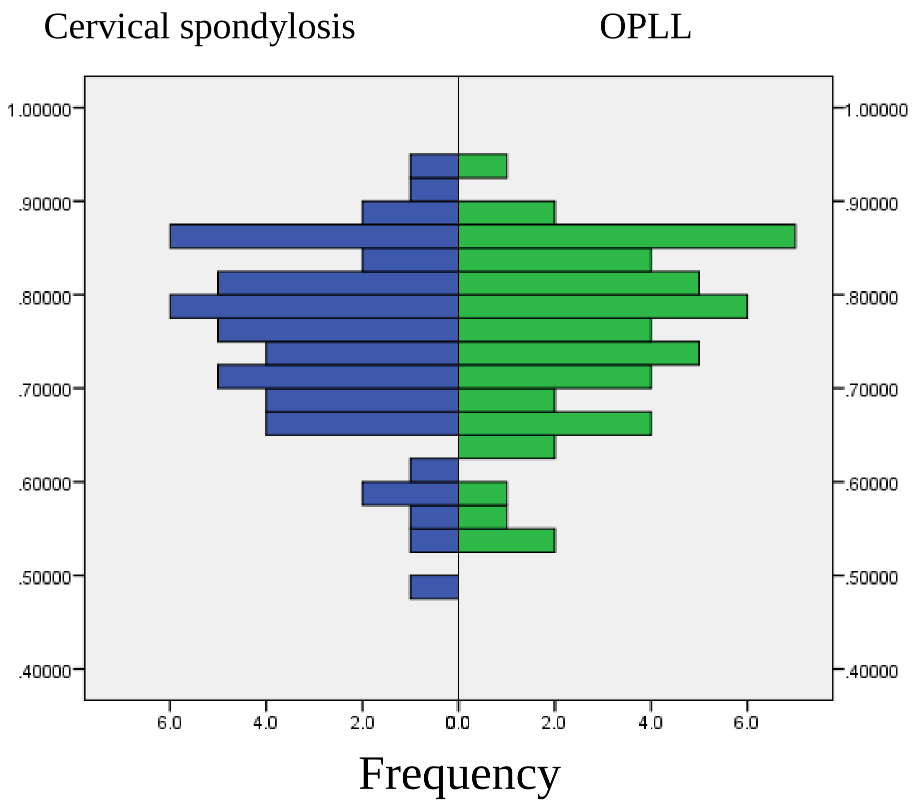

Supplementary figure 2. Histogram of propensity scores in the propensity score-matched cohort.

Supplemental Table 1. Scoring system for cervical myelopathy (C-JOA score)

I Upper extremity motor function

0: Unable to eat unaided with any type of eating utensil, including chopsticks, spoon, or fork and/or unable to fasten buttons of any size

1: Can eat unaided with spoon and/or fork but not chopsticks

2: Either eating with chopsticks or writing is possible but not practical and/or large buttons can be fastened

3: Either eating with chopsticks or writing is clumsy but possible and/or cuff buttons can be fastened

4: Normal

II Lower extremity motor function

0: Unable to stand and walk by any means

0.5: Able to stand but unable to walk

1: Unable to walk without a cane or other support on level ground

1.5: Able to walk without support but with a clumsy gait

2: Walks independently on level ground but needs support on stairs

2.5: Walks independently when going upstairs but needs support when going downstairs

3: Capable of walking fast but clumsily

4: Normal

III Sensory function

A. Upper extremities

0: Complete loss of touch and pain sensation

0.5: ≤50% normal sensation and/or severe pain or numbness

1: >60% normal sensation and/or moderate pain or numbness

1.5: Slight subjective numbness without any objective sensory deficit

2: Normal

B. Lower extremities

Same as A

C. Trunk

Same as A

IV Bladder function

0: Urinary retention and/or incontinence

1: Sensation of retention, dribbling, thin stream, and/or incomplete continence

2: Urinary retention and/or pollakiuria

3: Normal

Total score for normal = (I + II + III = IV) = 17

Recovery rate = (postoperative score minus preoperative score) ×100 / (17 minus preoperative score)

Supplemental Table 2. Japanese Orthopaedic Association Cervical Myelopathy Evaluation Questionnaire

With regard to your state of health during the last week, please circle the number of the answer for each of the following questions that best applies. If your condition varies from day to day or throughout the day, please circle the number when that item is at its worst.

Q1-1 While in the sitting position, can you look up at the ceiling by tilting your head upward?
1) Impossible. 2) Possible to some degree (with effort). 3) Possible without difficulty.

Q1-2 Can you drink a glass of water without stopping despite your neck symptoms?
1) Impossible. 2) Possible to some degree. 3) Possible without difficulty.

Q1-3 While in the sitting position, can you turn your head toward a person who is seated to the side but behind you and speak to that person while looking at his/her face?
1) Impossible. 2) Possible to some degree. 3) Possible without difficulty.

Q1-4 Can you look at your feet when you go down stairs?
1) Impossible. 2) Possible to some degree. 3) Possible without difficulty.

Q2-1 Can you fasten the front buttons of your blouse or shirt with both hands?
1) Impossible 2) Possible if I take my time. 3) Possible without difficulty.

Q2-2 Can you eat a meal with your dominant hand using a spoon or a fork?
1) Impossible. 2) Possible if I take my time. 3) Possible without difficulty.

Q2-3 Can you raise your arm? (Answer for the weaker side)
1) Impossible. 2) Possible up to shoulder level. 3) Possible although the elbow and/or wrist is slightly flexed. 4) I can raise it straight upward.

Q3-1 Can you walk on a flat surface?
1) Impossible. 2) Possible but slowly even with support. 3) Possible only with the support of a handrail, cane, or walker. 4) Possible but slowly without any support. 5) Possible without difficulty.

Q3-2 Can you stand on either leg without the support of your hand? (Without needing to support yourself)

1) Impossible with either leg. 2) Possible on either leg for more than ten seconds. 3) Possible for each leg individually for more than ten seconds.

Q3-3 Do you have difficulty going up stairs?

1) I have great difficulty. 2) I have some difficulty. 3) I have no difficulty.

Q3-4 Do you have difficulty in one of the following movements: bending forward, kneeling, or stooping?

1) I have great difficulty. 2) I have some difficulty. 3) I have no difficulty.

Q3-5 Do you have difficulty walking for more than 15 minutes?

1) I have great difficulty. 2) I have some difficulty. 3) I have no difficulty.

Q4-1 Do you have urinary incontinence?

1) Always. 2) Frequently. 3) Only when I have not passed urine for more than 2 hours. 4) When sneezing or straining. 5) No.

Q4-2 How often do you go to the bathroom at night?

1) Three times or more. 2) Once or twice. 3) Rarely.

Q4-3 Do you have a feeling of residual urine in your bladder after voiding?

1) Most of the time. 2) Sometimes. 3) Rarely.

Q4-4 Can you start your urine stream immediately when you want to void?

1) Usually not. 2) Sometimes. 3) Most of the time.

Q5-1 How do you rate your present health condition?

1) Poor. 2) Fair. 3) Good. 4) Very good. 5) Excellent.

Q5-2 Have you been unable to work or go about your normal activities?

1) I have not been able to do them at all. 2) I have been unable to do them most of the time. 3) I have sometimes been unable to do them. 4) I have been able to do them most of the time. 5) I have always

been able to do them.

Q5-3 Has your work routine been hindered because of pain?

1) Greatly. 2) Moderately. 3) Slightly (somewhat). 4) Little (minimally). 5) Not at all.

Q5-4 Have you been feeling discouraged or depressed?

1) Always. 2) Frequently. 3) Sometimes. 4) Rarely. 5) Never.

Q5-5 Do you ever feel exhausted?

1) Always. 2) Frequently. 3) Sometimes. 4) Rarely. 5) Never.

Q5-6 Do you feel happy?

1) Never. 2) Rarely. 3) Sometimes. 4) Almost always. 5) Always.

Q5-7 Do you think you are in decent health?

1) Not at all (my health is very poor). 2) Barely (my health is poor). 3) Not much (my health is average). 4) Fairly (my health is better than average). 5) Yes (I am healthy).

Q5-8 Do you feel your health will get worse?

1) Very much so. 2) A little bit at a time. 3) Sometimes yes and sometimes no. 4) Not very much. 5) Not at all.

Supplemental Table 3. Japanese Orthopaedic Association Back Pain Evaluation Questionnaire

With regard to your state of health during the last week, please circle the number of the answer for each of the following questions that best applies. If your condition varies from day to day or throughout the day, please circle the item number of your condition at its worst.

Q1-1 You often change your posture to alleviate low back pain
1) Yes. 2) No.

Q1-2 You lie down more often than usual because of low back pain
1) Yes. 2) No.

Q1-3 Your lower back is almost always aching
1) Yes. 2) No.

Q1-4 You cannot sleep well because of low back pain. (If you take sleeping pills because of the pain, select "No")
1) No. 2) Yes.

Q2-1 You sometimes ask someone to help you when you are doing something because of low back pain
1) Yes. 2) No.

Q2-2 You have stopped bending forward or kneeling down because of low back pain
1) Yes. 2) No.

Q2-3 You have difficulty in standing up from a chair because of low back pain
1) Yes. 2) No.

Q2-4 Because of the low back pain, turning over in bed is difficult
1) Yes. 2) No.

Q2-5 You have difficulty putting on socks or stockings because of low back pain
1) Yes. 2) No.

Q2-6 Do you have difficulty with any of the following movements: bending forward, kneeling,

or stooping?

1) I have great difficulty. 2) I have some difficulty. 3) I have no difficulty.

Q3-1 You walk only short distances because of low back pain

1) Yes. 2) No.

Q3-2 You stay seated most of the day because of low back pain

1) Yes. 2) No.

Q3-3 You go up stairs more slowly than usual because of low back pain

1) Yes. 2) No.

Q3-4 Do you have difficulty in going up stairs?

1) I have great difficulty. 2) I have some difficulty. 3) I have no difficulty.

Q3-5 Do you have difficulty in walking for more than 15 minutes?

1) I have great difficulty. 2) I have some difficulty. 3) I have no difficulty.

Q4-1 You do not do any routine housework these days because of low back pain

1) No. 2) Yes.

Q4-2 Have you been unable to do your normal work or perform activities of daily living as well as you would like?

1) I have not been able to do them at all. 2) I have been unable to do them most of the time. 3) I have sometimes been unable to do them. 4) I have been able to do them most of the time. 5) I have always been able to do them.

Q4-3 Has your work routine been hindered because of pain?

1) Greatly. 2) Moderately. 3) Slightly (somewhat). 4) Little (minimally). 5) Not at all.

Q5-1 You become irritated or angry with other persons more often than usual.

1) Yes. 2) No.

Q5-2 How is your present state of health?

1) Poor. 2) Fair. 3) Good. 4) Very good. 5) Excellent.

Q5-3 Have you been feeling discouraged or depressed?

1) Always. 2) Frequently. 3) Sometimes. 4) Rarely. 5) Never.

Q5-4 Do you ever feel exhausted?

1) Always. 2) Frequently. 3) Sometimes. 4) Rarely. 5) Never.

Q5-5 Have you been feeling happy?

1) Never. 2) Rarely. 3) Sometimes. 4) Almost always. 5) Always.

Q5-6 Do you think you are in good health?

1) Not at all (my health is very poor). 2) Barely (my health is poor). 3) Not really (my health is average). 4) Fairly (my health is better than average). 5) Yes (I am healthy).

Q5-7 Do you feel your health will get worse?

1) Very much so. 2) A little bit at a time. 3) Sometimes yes and sometimes no. 4) Not very much. 5) Not at all.

Supplemental Table 4. Visual analog scale for physical pain

Mark a point between 0 and 10 on the lines below to show the degree of your pain (numbness) when your symptom was at its worst during the last week (0 means “no pain (numbness) at all” and 10 means “the most intense pain (numbness) imaginable”.

JOACMEQ VAS score

Severity of pain or stiffness in your neck or shoulders

0 ______________________________ 10

Severity of tightness in your chest

0 ______________________________ 10

Severity of pain or numbness in your arms or hands

0 ______________________________ 10

Severity of pain or numbness from the chest to the toes

0 ______________________________ 10

JOABPEQ VAS score

Severity of low back pain

0 ______________________________ 10

Severity of pain in the buttocks and lower limbs

0 ______________________________ 10

Severity of numbness in the buttocks and lower limbs

0 ______________________________ 10

JOA, Japanese Orthopaedic Association; JOACMEQ, JOA Cervical Myelopathy Evaluation Questionnaire; JOABPEQ, JOA Back Pain Evaluation Questionnaire; VAS, visual analog scale
